# Establishing the acceptability and usability of an animated virtual patient simulation

**DOI:** 10.1016/j.rcsop.2021.100069

**Published:** 2021-09-08

**Authors:** Charlotte Lucy Richardson, Stephen Chapman, Simon White

**Affiliations:** a School of Pharmacy, Faculty of Medical Sciences, Newcastle University, NE1 7RU, UK; b School of Pharmacy and Bioengineering, Keele University, ST5 5BG, UK

**Keywords:** Virtual patient, Simulation, Counselling, Education, Pharmacist, CPD, continuing professional development, IQR, interquartile range, NOAC, non-vitamin K oral anticoagulant, VP, virtual patient

## Abstract

**Background:**

An animated, video-based, virtual patient (VP) has been developed to allow pharmacists to learn how, and practice how, to advise patients taking non-vitamin K oral anticoagulants, a group of high-risk medicines. VPs are well-established resources but have historically only been accessed within specific online teaching sessions or at university sites; this new VP represents a mobile design that can be accessed from anywhere.

**Objective:**

To investigate the usability and acceptability of the VP application with a focus on exploring perspectives on accessibility.

**Methods:**

The study used an exploratory sequential mixed method design consisting of a satisfaction survey and interviews. Survey data were analysed descriptively to assess satisfaction with the application and to identify interview discussion areas. Interview data were analysed using the Framework Approach to thematic analysis. Participants were hospital or community pharmacists, or pre-registration pharmacists.

**Results:**

A total of 94 survey responses were collected and 22 respondents went on to take part in an interview. Participants reported liking the concept and delivery of the VP, particularly the high-quality technology. They also reported finding it usable, and appeared to favour its mobility and accessibility, particularly as the VP can be used on any internet accessible device, including mobile phones, with no specific requirements. Amendments that were suggested included quickening the delivery of some animations and improving navigation within the application, possibly through a button to return to the previous step should a mistake be made.

**Conclusions:**

The mobile VP appeared to be functional and usable, with the majority of users reporting satisfaction with use across a range of devices. Users reported positively about the VP's remote access, but navigation around the application requires development.

## 1 Introduction

Virtual patient (VP) technology is an evolving method of simulation that has benefits in delivering remote experiential learning across health professions.^1^, ^2^, ^3^ Good user satisfaction has been reported in previous studies that have evaluated both older and newer types of VP technologies.^4^, ^5^, ^6^ However, VPs have been defined in various ways, which is reflective of their many uses, particularly as there are various ways of designing them and VP technology can incorporate various modalities such as animation, voice recognition and video technology.^7^ Therefore, for clarity, the following definition of VP was adopted in this study^8^^,^^9^:

“A virtual patient is an interactive computer simulation of a computer programmable patient (or avatar) in a real-life clinical scenario for the purpose of medical training, education, or assessment that will respond to learner decisions.”

VPs are usually highly individualised tools that incur significant design costs, and so historically, they have only been accessible online from links in specific courses, such as degree programmes and modules, or they have been developed as pieces of equipment that require dedicated physical teaching spaces. Even the online types of these VPs have often required particular software or hardware for use.^10^ This has meant that the accessibility of such resources has been limited and there is a lack of VPs that are more widely usable and accessible within the pressures of daily professional practice and clinical environments.^11^^,^^12^ Increasing the accessibility of VPs seems germane, given the broad move towards increased use of technology for health professional education, training and continuing professional development (CPD).^10^^,^^13^, ^14^, ^15^

Non-vitamin K oral anticoagulants (NOACs) are deemed to be ‘high risk medicines’,^16^^,^^17^ which require extra advice and support to be given to patients about taking the medicine and on the risks and benefits of use.^18^^,^^19^ To our knowledge, VPs have not previously been developed that concern NOACs, although other VP programmes are available for broader topics such as ‘medication reviews’ and also for some formulations of medicines, such as inhaled formulations. At present, pharmacists may undertake additional learning or CPD on NOACs use, but typically this is factual and clinical, usually without applying the knowledge or practicing how to communicate this information to patients. A VP programme was developed to offer the opportunity to learn the same clinical information relating to NOACs, but in such a way that the pharmacist can see, experience and practice communicating in a patient interaction. In this paper the evaluation of the VP is discussed in terms of its design, functionality, and perceived acceptability and usability, rather than its educational merit which will be reported elsewhere.

### 1.1 Aim

The aim of this study was to investigate the usability and acceptability of a new style of VP; explore the perspectives of pharmacists and pre-registration pharmacists on the VP's accessibility; and identify ways in which the VP can be improved.

## 2 Methods

### 2.1 The VP design

In this study the VP was developed to be mobile (it is accessed via a purpose-built website) and widely accessible (there are no specific or specialised requirements for use, only log in details and an internet connection) for use in education and training courses or for individuals to use as part of their CPD. A learning topic was chosen that was well-suited to experiential learning (i.e. applying knowledge gained and practicing skills rather than purely knowledge increase) and on a clinically important medicines-related topic to maximise benefit for patients (NOACs).

**Fig. 1:**
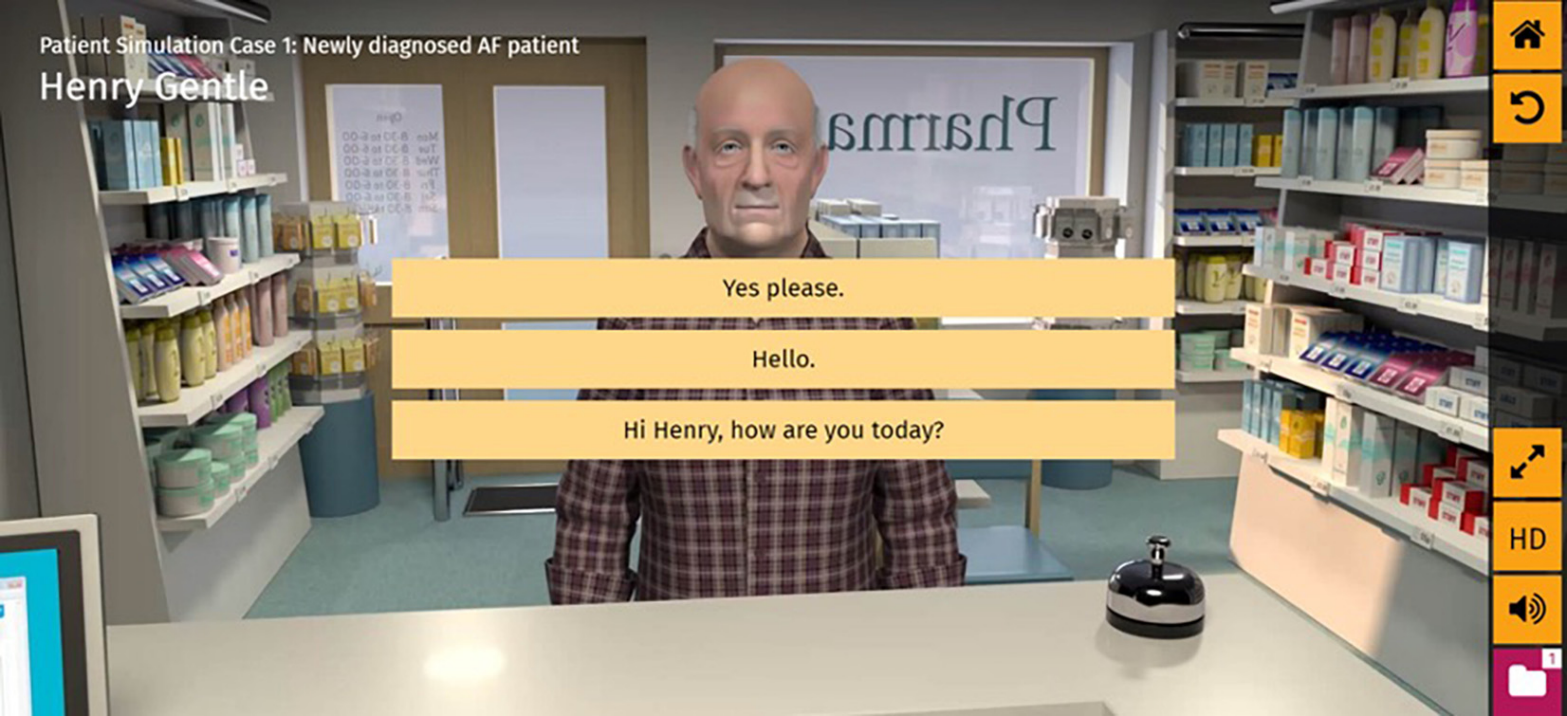
The virtual patient programme interface.

The VP application was developed by Keele University and Bayer AG^20^ and it aims to teach the user (pharmacists or pre-registration pharmacists; pre-registration trainees are trainees within the UK that are in a training year between graduation from university and qualification to the pharmacist register) how to advise patients on safely taking NOACs (Fig. 1.). The intended learning outcome of the application was that after having worked through the VP programme, users should be able to: *“demonstrate how to advise a new patient on rivaroxaban”*. The VP used sophisticated, animated video technology to deliver a simulation that aimed to develop consultation skills, where specific but tailored information should be effectively given to a patient in a way that accommodates their needs.

The design used a decision tree to present multiple choice options to the user for them to steer the consultation in the way they wanted. The VP consisted of computer-generated, animated, videos that played a relevant animation, depending on the decision made. The systems used *Hypertext Mark-up Language* (HTML), *cascading style sheets* (CSS) and *JavaScript* video renders that allowed the VPs to work on mobile phones, tablets, and desktop devices.

At the end of using the programme, the user received personalised feedback on their performance in terms of both factual-clinical aspects and communication skills. Users were free to use the programme as many times as they wished, but it was anticipated that use of the VP from start to finish would, as a minimum, take 15 min. However, users' actual duration of use would depend on factors such as their style of use, their available time, and their foundation of knowledge on the topic.

### 2.2 Study design

The study used exploratory sequential mixed methods, which involved conducting a satisfaction survey followed by qualitative semi-structured interviews. This design was novel compared to previous VP evaluations, since the majority of previous VP studies have used quantitative methods alone.^21^, ^22^, ^23^, ^24^ The semi-structured interviews with consenting survey respondents were intended to further explore and contextualise the survey results, particularly unexpected findings.

### 2.3 Recruitment and participation

The interview sample was estimated to be 15–25 participants based on the homogeneity of the sample and the small number of characteristics relevant to the study aim.^25^ Based on the need to recruit a large enough sample for the interview phase, the target sample size for the survey was estimated to be 80–100 respondents using a 20–25% sequential approach to sampling^26^; Use of a sample size calculation was not appropriate. Ethical approval from Keele University (ERP1361) and UK Health Research Authority approval (Ref 23,513) was obtained.

Participants had to be practising pharmacists or pre-registration trainee pharmacists working at one of the study sites (*n* = 14). Sites covered geographical regions of community pharmacies or hospital trusts across England. The study was promoted via UK Clinical Research Networks to recruit a mixture of sites from across England. Identification and recruitment of individual participants from these sites used a mixture of email, social media and word of mouth to identify a range of potential participants. There was no monetary incentive to take part other than to gain access to a novel educational tool which offered a CPD opportunity.

Consenting participants were initially recruited to the survey phase of the study where they were provided with access to an online survey that included access to the VP and instructions for use. As part of the survey, participants were asked for their interest in taking part in a follow up interview and following the survey phase of the study a varied group were followed up and interviewed. Interview participants were selected based on their range of views including selecting those with responses which deviated from the majority of responses within the survey, and based on respondent demographics (e.g., age, gender and sector of practice) to obtain a varied sample for the interview phase. This was possible as one researcher (CR) had access to deanonymized consent form information that enabled identification of participants for follow up. Interview participants were contacted around one week prior to the interview, provided with access to the VP and asked to re-familiarise themselves with it before the interview.

### 2.4 Data collection

The satisfaction survey was a subsection of a wider questionnaire exploring a range of factors associated with use of the VP. The satisfaction survey was made up of Likert questions addressing the VP's acceptability and usability to the participants. These questions captured the VP‘s perceived usefulness, enjoyment in use, difficulty in use, comfort in use, likeliness to use again and likeliness to recommend it to others. The survey also asked a series of questions including about respondents’ demographics. Where possible, satisfaction questions (5-point Likert scales) from other VP evaluations were used to contribute to the quality and rigor of the instrument.^5^^,^^14^^,^^21^^,^^24^^,^^27^^,^^28^ No complete pre-existing tool could be identified for use in this study. The tool was piloted with 12 participants prior to use to assess its understandability and ease of completion; minor amendments to the wording of some questions were made following this.

The interviews used a semi-structured approach to further explore the use of the VP in practice and were conducted via a mixture of media namely, face to face, telephone and video calling. The interviews used an interview guide that was iterative as interviews progressed (supplementary file 1). The interviewer (CR) was trained to conduct interviews and underwent a process of reflexivity throughout the research. This included discussing and reflecting on the process and conduct of interviews and the analysis with other members of the research team, particularly in relation to ways that the research team's characteristics may have affected the construction of the data; a reflexive diary was also kept by CR for this purpose.

### 2.5 Data analysis

Survey data were anonymised by the lead researcher (CR), which involved assigning each participant an identification number and then deleting personally identifiable information from the database of responses used for analysis. The data were initially analysed using descriptive statistics, which involved calculations of medians and interquartile ranges (IQR) to consider the average and spread of the data across the Likert scales; means were not used due to the ordinal nature of the data.

Interviews were audio-recorded and transcribed verbatim before being analysed using the Framework Approach to thematic analysis to identify themes within the data.^29^, ^30^, ^31^ The analysis followed the stages of the framework approach as discussed by Pope et al. and Gale et al., including familiarisation with the data, coding of the data, development of a framework, and mapping of the codes to the framework.^30^^,^^31^ From the framework that was developed, emergent themes were identified and refined by comparison with other themes and the transcripts, and discussion between members of the research team about meanings in the data.

## 3 Results

### 3.1 Survey

A total of 94 participants completed the survey between November 2018 and August 2019 (Table 1). This included participants from hospital (*n* = 61), community (*n* = 26) and other (*n* = 7); and of pre-registration pharmacists (*n* = 23) and registered pharmacists (*n* = 71). Further analysis was undertaken to explore possible differences between those from the hospital versus the community sector and between pharmacists and pre-registration pharmacists, but no such differences were identified.

**Table 1:** Respondent demographics.

	Whole sample	Pre-registration pharmacists	Registered pharmacists	Community	Hospital
Gender					
Male	24 (25.5%)	1 (4.3%)	23 (32.4%)	8 (30.8%)^⁎^	13 (21.3%)^⁎^
Female	70 (74.5%)	22 (95.7%)	48 (67.6%)	18 (69.2%)	48 (78.7%)

Age group					
20–29 years	48 (51.1%)	22	26	11	33
30–39 years	29 (30.8%)	0	29	8	18
40–49 years	13 (13.8%)	1	12	4	9
50–59 years	4 (4.3%)	0	4	3	1
Older than 60 years	0 (0%)	0	0	0	0

Qualification status					
Pre- registration pharmacists	23 (24.5%)			3	18
Registered pharmacists	71 (75.5%)			23	43

Sector of practice					
Community	26 (27.7%)	3	23		
Hospital	61 (64.8%)	18	43		
Other (e.g. general practice)	7 (7.4%)	2	5		

**Fig. 2:**
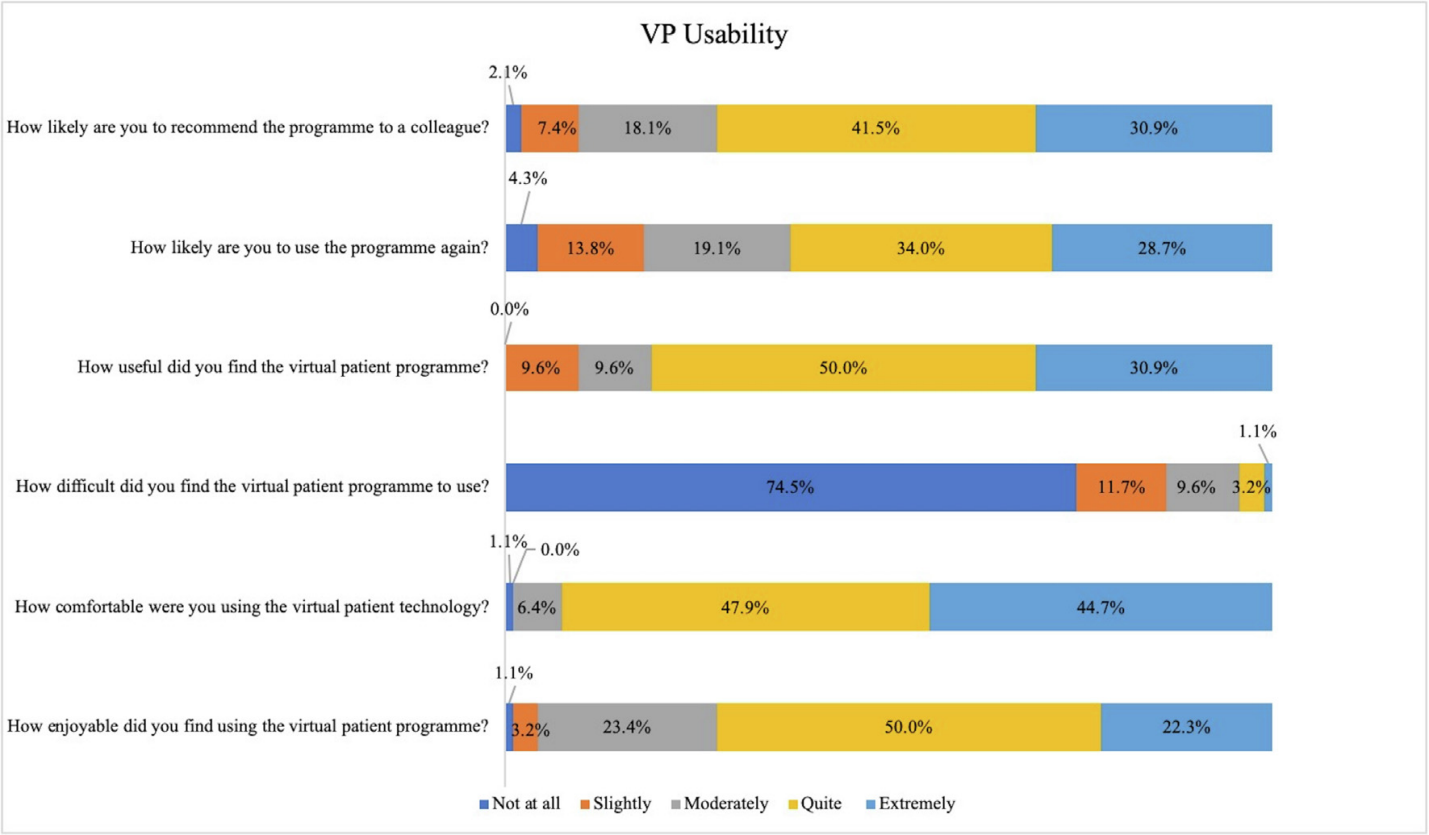
VP usability accessed via six questions each question is across a Likert of not at all to extremely.

The Likert questions regarding satisfaction and the VP's usability (Table 2. And Fig. 2.), used a scale of 1 “not at all” to 5 “extremely”. For the questions regarding enjoyment, comfort, usefulness, likeliness to use again and likeliness to recommend it, the median scores were “quite” (median 4; IQR = 1, except questions concerning repeated VP use and recommending it where IQR = 2).

**Table 2:** Median responses to questions relating to the usability of the virtual patient programme, measured on a scale of 1 “not at all” to 5 “extremely”.

Question (all measured on a Likert scale of 1 not at all to 5 extremely)	Median	IQR
How enjoyable did you find using the virtual patient programme?	4 - quite	1
How comfortable were you using the virtual patient technology?	4 - quite	1
How difficult did you find the virtual patient programme to use?	1 – not at all	1
How useful did you find the virtual patient programme?	4 - quite	1
How likely are you to use the virtual patient programme again?	4 - quite	2
How likely are you to recommend the virtual patient programme to a colleague?	4 - quite	2

Despite the same median (median 4), there were some differences in the spread of results across the Likert scales (Fig. 2.). Enjoyment using the VP had 73.4% of responses for “moderately” or “quite”. The question regarding the usefulness of the VP was more positive as 80.9% of responses were for Likert scores of “quite” and “extremely”. For the question regarding difficulty using the VP, the median score was “not at all” (IQR = 1), suggesting that the majority of respondents (86.2% for “not at all” and “slightly”) did not find the technology difficult to use. These usability questions indicated that respondents, found the VP usable and would use it again and/or recommend it (72.4% agreed or strongly agreed they would recommend to a colleague; 62.7% agreed or strongly agreed they would use again).

### 3.2 Interview results

Interviews provided further insights by directly exploring the results of the satisfaction survey with participants. A total of 22 interviews took place with an average duration of 31 min (Range 19–43 min). Two themes relating to usability and the VP technology emerged from the data: ‘running of the VP’ and ‘technological improvements’ (see supplementary file 2 for further detail).

#### 3.2.1 Running of the VP

The VP technology appeared to be functional, and most users reported good access and usability although a minority of participants did report encountering varying technological issues. These included problems accessing and running the VP, although it was not clear in some cases why this had occurred.

“So, the first time I did it, it was absolutely fine. Er, it worked like a treat. Erm, however, when I... went back on that same computer to use it, it did freeze, and it kept freezing which was frustrating...”

[P35, hospital pharmacist]

Some participants also reported parts of the VP being slow, which appeared not to be due to the technology, but rather the design and the speed at which the application had been programmed to deliver the animated responses.

“I think it was more that the patient was talking slow and…everything else was fine and the whole programme went fast and everything….”

[P50, hospital pre-registration trainee]

The next most common discussion area concerned access to the VP. This was not raised by all participants, but of those that did, all said that access to the VP was at least adequate.

A group of participants accessed the VP via a mobile telephone, these participants spoke in overwhelmingly positive terms about this mobility.

“I thought it was good that it works on my mobile. I didn't think it would.”

[P57, hospital pharmacist]

Furthermore, a number of participants explicitly expressed that they thought the VP was user-friendly.

“Yeah, I mean because it's digital, you can use it anywhere really, bottom line…I think it's fantastic. I think I love how you can get it up on different devices, like iPads.”

[P17, mixed hospital and general practice pharmacist]

#### 3.2.2 Technological improvements

All participants suggested some form of technological amendment to improve the user experience. These ranged from minor adjustments where the experience was thought to not be optimum, to more significant changes that could affect technological delivery of the VP. However, most participants appeared to have still found the VP to be useable without the suggested changes being made.

“I think it's [the VP] a very good idea… I'm highlighting issues that it can improve...It's constructive criticism”

[P35, hospital pharmacist]

A further example concerned improvements to the speed of the patient talking to minimise the previously discussed ‘slow animations’.

The technological change most frequently discussed was the introduction of a ‘back’ button so that the user could return to previous decision points within the application.

“I don't know if there was an option to go back and change your option. I feel like there wasn't so if you had made that decision you [were not] able to go back.”

[P5, hospital pre-registration pharmacist]

For those who thought a back button was a good idea, the main reasons appeared to be because they could attempt to rectify the consultation if they thought it was going less than optimally or because they accidently clicked an option they had not intended to. This was a more common perspective from pre-registration pharmacists.

“I clicked something by accident at one point, but I wasn't able to go back and just change it. I had to start it all again.”

[P5, hospital pre-registration pharmacist]

On the other hand, some participants said that use of a back button was not a good idea. The commonest reason for this was that there is no such thing as a ‘back button’ in real-life practice.

“It would be useful if you could go back. But in real life, if you start a consultation and you said something or you make a decision, how do you backtrack? Like you can't really, or you just have to rectify it as you progress. So, I think – well I suppose from the user-friendly point of view it would be nice to have as an additional option, but in reality, it doesn't happen.”

[P74, community pharmacist]

## 4 Discussion

The majority of respondents appeared to find the VP acceptable and usable, with few technical limitations. The VP functioned as a mobile and accessible application available without any specific requirements other than an internet connection. The few reports of freezing or trouble loading the VP were more likely to have been due to the user's internet connection or the device used than the VP software, according to the expert technical review of the software by the VP's programmers. However, some design choices have been identified as needing optimisation such as the speed of animation and potentially the use of a ‘back’ button.

Some studies have previously incorporated VP access via common web browsers, but no applications appear to have considered the consequences of this for use on mobile devices and how this could affect VP access.^14^^,^^32^ This is in part because VPs have previously not been accessible outside of defined locations and so mobility has not been a widely documented discussion area for VPs.^23^^,^^33^ Connectivity and internet access will also need further investigation as technologies are developed to be used at home or on the move. Similarly, the animations, although particularly well-received for being lifelike, may benefit from running at a faster speed, as this detracted from usability for a minority of participants. However, this limitation may have been heightened by variable internet connections.

A number of participants highlighted that they thought that the VP was user-friendly. This finding differs from previous work that found that it may take users a period of time to become accustomed to using the technology, during which they may not find it to be user-friendly.^14^ This could also be reflective of increased use of technology in all parts of life. The participants did not appear to have to adjust to using this VP which is encouraging as Taglieri et al., pointed out that familiarity with the technology can improve learning.^33^

Despite this, a small number of users suggested altering the VP's interface to create a ‘back button’ to allow for better navigation within the patient case. This was more common in pre-registration pharmacists, possibly because of their lack of experience of the topic area relative to qualified pharmacists so they may have been more likely to make ‘mistakes’ within the consultation and want to go back a step. This may improve the user experience but, from an educational point of view the use of a back button may not be in keeping with one of the recognised advantages of VPs, which is being able to safely experience the consequences of mistakes.^8^^,^^34^ Additionally, a back button may reduce the realism of the experience as there is no such thing as a ‘back button’ in real life; something which was directly identified by some participants. The current lack of a back button does not appear to be prohibitive of VP use but improved navigation within the system does need to be considered and in such a way that embraces the triad of the: educational purpose of the application which includes it being a safe space to experience the consequences of mistakes; the realism and authenticity to practice; and technical usability.

The VP uses experiential learning^35^^,^^36^ to cyclically develop skills in NOAC counselling. It is possible that the mobility of the VP contributed to bringing this learning closer to clinical practice as the VP could be accessed at any time, following self-identification of areas for development as part of routine CPD or following critical practice-based incidents.^37^ Mobile learning resources are recognised to be able to bridge the gap between classroom and practice^12^ although this appears to be the first time that the acceptability of a VP has been evaluated to consider mobility and mobile phone access.

The VP technology and the novel, mobile device access, have applicability across health professions as we move towards lifelong continuous CPD alongside an increasingly pressurised health environment where technology needs to be complimentary to and accessible within the working behaviours of the users. The VP has demonstrated that it is possible to design an online, remotely accessed, usable simulation and this has transferability to various sectors and professions. Indeed, the COVID-19 pandemic in particular has since seen options for face-to-face role play as a simulation opportunity become highly limited and healthcare training has subsequently looked to utilise blended learning methods, which has increased the need for more readily accessible online tools such as this VP.^38^^,^^39^

### 4.1 Limitations

This study focuses on the technical usability of a single, mobile learning VP and the findings may not necessarily be transferrable to other mobile learning VPs. Similarly, VP applications on different clinical topics may not be experienced as favourably by users. However, whilst VPs can be highly specific technologies, the description and detail provided here is intended to facilitate assessments of such similarities and transferability of findings to better design and implement future applications. It is also acknowledged that the survey sample size may not have been representative of the whole population eligible to participate, and that participant recruitment was lower in the community sector (*n* = 26) than the hospital sector (*n* = 61) for both study phases. This could be seen as a limitation but, as highlighted, there were no apparent differences between the community and hospital groups during data analysis.

## 5 Conclusion

The VP technology was functional and well-liked by participants, and a novel feature of mobile access has been demonstrated. Some design features, such as navigation around the application need development to better improve user experience. This study can help to contribute to identifying an ‘ideal’ VP design that is both usable and accessible yet educationally valuable.

## Authorship

Charlotte Richardson, Professor Stephen Chapman, and Dr. Simon White planned, designed and drafted the manuscript. Charlotte Richardson also conducted the analysis for the manuscript.

## Declaration of Competing Interest

The VP was commissioned and paid for by, Bayer AG. They had no involvement in the design or conduct of the research nor in the drafting of this manuscript. There are no competing interests concerning this study. Henry Gentle, the virtual patient character is not an actual patient. Any resemblance to real person living or deceased is a coincidence.

## Appendix A Supplementary data

Semi-structured interview guide Image 1

Themes and example quotes from semi-structured interviews Image 2
