# Supplementary material for: Establishing the acceptability and usability of an animated virtual patient simulation
Source: Explor Res Clin Soc Pharm. 2021 Sep 8;4:100069. doi: 10.1016/j.rcsop.2021.100069 (PMC9031081; doi:10.1016/j.rcsop.2021.100069)
Supplement: Supplementary file 1 — Semi-structured interview guide [file mmc1.docx]

Interview guide
*An Evaluation of the use of Virtual Patient Technology in
Pharmacist Education on Atrial Fibrillation*

Overview

1. Greetings, welcome, explanation of the structure of the session and the interviewer’s role.
2. Confirmation of informed consent and that the participant has used the VP intervention.
3. A semi-structured interview see guide below.
4. Summary and closing remarks. Opportunity for further questions from the participant. Contact details and dissemination of the study results highlighted. Thanks.

Interview Plan

a) Tell me about your current and past roles in pharmacy.

b) Experiences of AF and NOACs

- What are your experiences to date of NOACs and AF?

*Probe: What about the patients with AF on NOACs?*

- Tell me your thoughts and feelings on education and counselling for pharmacists on NOACs in AF

*Prompt: What are your experiences of education and training for NOACs in AF?*

*Probe: Do you think more education and training is needed?*

c) Experiences of virtual patients

- What are your experiences to date of VPs?

*Prompt: Prior to today, had you heard of/experienced/used VPs? When and what for? Did you have a good or bad experience?*

- What are your feelings towards using VPs? …… And why?

d) VPs as an educational tool in Pharmacy

- What are your thoughts on using VPs as an educational tool in pharmacy?

*Probe: What about specifically for AF and NOAC education and training?*

*Probe: How does the use of VPs compare to other E&T methods?*

*Probe: What are your feelings towards VP use in practice?*

*Probe: What sort of impact do you think the VP intervention could have on patients?*

e) VPs: The Technology

- Describe your thoughts regarding the intervention program

*Probes: Is there anything you want to discuss about the program,*

*Are there any good points or advantages you want to highlight?*

*Are there any improvements you can suggest?*

*Are there any negative points or disadvantages to raise?
Are there any changes you would make to the program?*

- Do you have any further comments regarding the technology, case or program?

- What are your feelings towards the VP technology and case?

- Do you think that by using the program you are better prepared to counsel NOAC taking AF patients? …. Why?

- Do you think that you are more likely to conduct these counsellings? …. Why?

*Prompt: Do you think your practice will change as a result of using the program?*

f) Further comments

- Is there anything else you would like to tell me or discuss?

*Prompt: Any other comments you wish to add or any further feedback on the program?*

- Are there any questions you would like to ask me?
