# Supplementary material for: Establishing the acceptability and usability of an animated virtual patient simulation
Source: Explor Res Clin Soc Pharm. 2021 Sep 8;4:100069. doi: 10.1016/j.rcsop.2021.100069 (PMC9031081; doi:10.1016/j.rcsop.2021.100069)
Supplement: Supplementary file 2 — Themes and example quotes from semi-structured interviews [file mmc2.docx]

| **Theme** | **Participant details** | **Quote** |
| --- | --- | --- |
| ***Running of the VP*** | 11, hospital pharmacist | *‘’Yeah, well I used it on an NHS [national health service] desktop, so it wasn’t particularly modern, so it worked fine, yeah, I didn’t have any difficulties.’’* |
|  | 14, hospital pharmacist | *‘’No, I think that was more or less it, it was user friendly, it was quite straightforward to actually use the programme.’’* |
|  | P50, hospital pre-registration trainee | *‘’The actual programme was fine.  It, it ran perfectly.’’* |
|  | 58, hospital pharmacist | *‘‘’It ran - yeah, it ran fine on the computer.  The only bit where it went a bit slow was during the feedback and I - it just - I just had to minimise it and bring it back up again and then it started running again but otherwise, it ran fine.’’* |
|  | 58, hospital pharmacist | *‘’Yeah. Um, yeah, I didn’t really have any issues, um, with kind of I wasn’t sure where to go from here. It was really smooth, you know, smooth running. You knew exactly what to do when sort of thing.’’* |
|  | 77, hospital pharmacist | *‘’Yes, yeah, there was no problem with speed or anything.’’* |
|  | P74, community pharmacist | *‘’I had no issues, it was very self-explanatory, it was easy to use.’’* |
|  | 83, community pharmacist | *‘’Usability I think um, yeah, I mean I found it very easy, really straightforward really, even from coming at it from my perspective.  It was intuitive in that respect um, and realised I had to click on responses and that would need further responses, and the, and to be fair, the err, you know, the patient or the character behaved err, in a way that wasn’t surprising to me, it was, it was quite interesting to see the responses seemed to be following my um, choices and so on.  And it, it wasn’t, it didn’t seem extreme or far-fetched, the response, the response seemed reasonably natural in, in the nature of the, such an activity, yeah, so I was, I was, you know, I was quite impressed by the, by the interaction, let’s say.’’* |
|  | P17, mixed hospital and general practice pharmacist | *‘’Um, yeah it was just very new and very different um, probably slightly off track but it looks a little bit like The Sims, the video game I would play when I was younger.’’* |
|  | P50, hospital pre-registration trainee | *‘’Yeah, I think it was better….I just quickly did it erm, when the pharmacy was a bit quiet.’’ [P50] [Response when asked about using the VP via a mobile telephone].* |
| ***Technological Improvements*** | P50, hospital pre-registration trainee | *‘’maybe have the patient speaking a bit faster and have the subtitles coming as he's speaking.’’* |
|  | P35, hospital pharmacist | *‘’Erm, for me, it was, like I said, something different, something new. It was - and it was exciting. However, it was almost pre-set and, like you said, algorithmic where it's just almost like - 'Okay, this gives this, and this gives this.' Erm, the concept and the idea is very good. It, it needs to be worked on. It needs to be tailored.’’* |
|  | 14, hospital pharmacist | *‘’It would be good if it could be personalised a bit as well so if it was err, erm, the patient that was being counselled initially if the pharmacist has to enter their name and so when the character, whoever the virtual person is talking to them, they can address them by name, personalising things.’’* |
|  | P74, community pharmacist | *‘’In terms of it from a learning perspective, probably. But I suppose if you had an option or some sort of – I don’t know, like a flag half way through to say ‘Are you sure about this?’ or ‘Are you happy with your choices? Do you want to change your decision or your advice on a certain matter?’, but that’s probably quite difficult to do from an IT perspective.’’* |
|  | 6, hospital pre-registration pharmacist | *‘’Yeah, I think so ‘cause I, I can’t, I can’t remember exactly when but at some point I was like, ‘Hmm, maybe I should have’, I can’t remember what option but I changed my mind but then I kind of, I did it twice I think.  I went all the way back just to get…’’* |
|  | 75, hospital pharmacist | *‘’I don’t know. I don’t think it’s anything major that needs amending. I think there may be a few points to maybe kind of have as a summary, um, about maybe a bit more feedback as to why certain options might be the case with certain patients, um, and maybe like highlighting the key points, um, to the consultation but I don’t think it’s anything that requires a lot of work.’’* |
